# Melanoma of Unknown Primary With Metastasis to the Brain: A Case Report and Review of the Literature

**DOI:** 10.7759/cureus.67022

**Published:** 2024-08-16

**Authors:** Tyler E Rice-Canetto, Drew Richard, Grace Kim, Ajay Ramnot, Javed Siddiqi

**Affiliations:** 1 Neurosurgery, Arrowhead Regional Medical Center, Colton, USA; 2 Neurosurgery, California University of Science and Medicine, Colton, USA; 3 Family Medicine, California University of Science and Medicine, Colton, USA; 4 Oncology, California University of Science and Medicine, Colton, USA; 5 Neurosurgery, Desert Regional Medical Center, Palm Springs, USA; 6 Neurosurgery, Riverside University Health System Medical Center, Moreno Valley, USA

**Keywords:** primary malignant melanoma of the lung, melanoma of unknown primary, skin melanoma, primary malignant melanoma, malignant melanoma metastasis

## Abstract

We present the case of a 72-year-old male found to have melanoma of unknown primary (MUP) in the lung with brain metastasis. The patient has a history of prostate cancer with radical proctectomy in 1999, hypertension with right-sided heart failure, and bilateral cataracts treated operatively. He presented to their home hospital after an unwitnessed fall, with a history of left-sided weakness. He was found to have a parietal lobe mass and two lung masses, where he was transferred to our hospital for a higher level of care. Biopsy of the lung lesion revealed melanoma, and the patient did not have any skin or mucosal foci present to indicate a primary source. We present this case in conjunction with a review of the literature. Following the Preferred Reporting Items for Systematic Reviews and Meta-Analyses (PRISMA) guidelines, our review resulted in 31 MUP case reports. Data was extracted on epidemiology, clinical presentations, diagnostics, treatment, and outcomes. The mean age was 57.5 with a male-to-female ratio of 1:1.3. The greatest instances of MUP occurred in prior smokers and patients with comorbidities, accounting for 17.95% of cases each. Thirty-one percent of patients presented with a growing palpable mass, 21% with gastrointestinal symptoms, and 21% with B-symptoms. Biopsy was the diagnostic standard, and the majority of patients also underwent biomarker studies. Treatment varied widely, and many patients underwent multiple phases. Outcomes ranged from death within several months to a disease-free period of three years. Our paper highlights the complexity and nuances of diagnosing MUP and primary malignant melanoma of the lung (PMML) and calls for further investigations to improve diagnostic and therapeutic approaches for rare presentations of melanoma. Despite limitations in sample size and data heterogeneity, this study highlights the diverse presentation and disease course of MUP, necessitating further studies to optimize patient outcomes.

## Introduction

Melanoma is a highly aggressive malignant tumor, most often arising from uncontrolled skin melanocyte growth [1]. In some instances, it arises from visceral organs, lymph nodes, or mucosal sites, such as the esophagus, liver, vagina, or oral cavity [2]. When melanoma is discovered extracutaneous without skin foci, it is melanoma of unknown primary (MUP), accounting for 3.2% of all melanomas [3]. Rarely, melanoma may originate in the lungs, termed primary malignant melanoma of the lung (PMML). This accounts for only 0.01% of primary lung neoplasms, with less than 45 reported cases [4]. We present the case of a 72-year-old male with an incidental finding of melanoma of the lung, without an identified skin origin. This case is presented with a review of the literature.

## Case presentation

A 72-year-old man presented to the hospital after an unwitnessed fall. Medical history included prostate cancer with radical proctectomy in 1999, hypertension with right-sided heart failure, and bilateral cataracts treated operatively. On admission, he reported left-sided weakness for several weeks. A computed tomography (CT) scan of the head revealed a 2.5 cm cystic lesion in the right parietal lobe. The patient was stabilized for transfer to our hospital. On arrival, he was started on Keppra 1 g twice daily and Decadron 6 mg every six hours.

The patient was worked up for metastatic cancer, and CT of the chest/abdomen/pelvis with contrast revealed two masses in the apical right lower lung lobe and a left kidney cyst. Given the patient's history of prostate cancer, total prostate-specific antigen was measured but was found to be <0.014 ng/ml. The patient was alert and oriented ×4, with a Glasgow Coma Score of 15 and no focal neurological deficits. 

**Figure 1:**
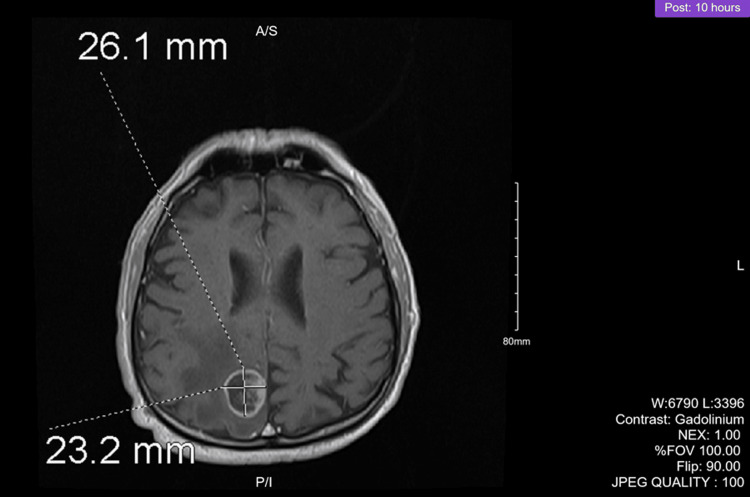
Preoperative brain MRI coronal view MRI: magnetic resonance imaging

**Figure 2:**
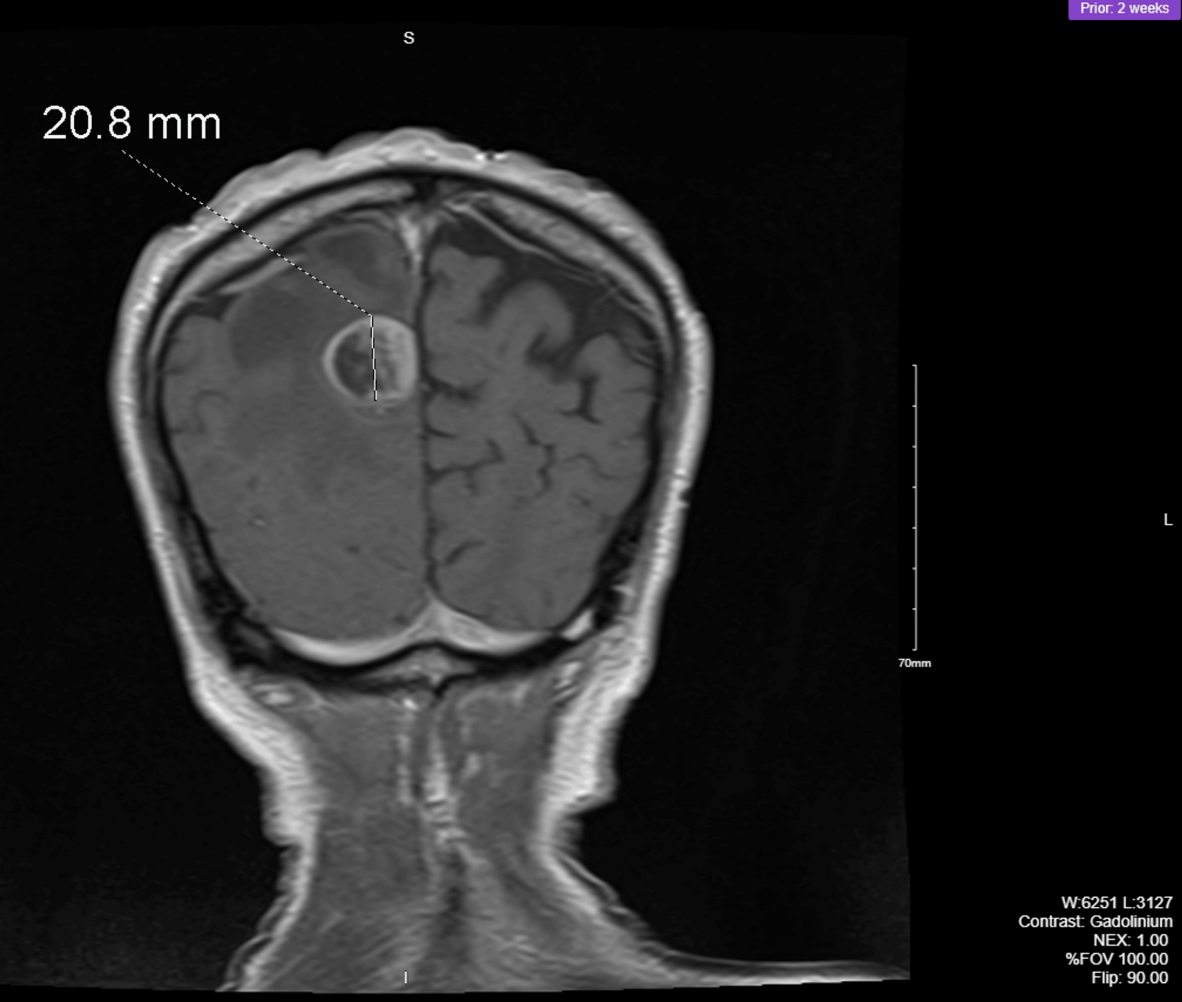
Preoperative brain MRI sagittal view MRI: magnetic resonance imaging

Figure 1 and Figure 2 illustrate the magnetic resonance imaging (MRI) of the brain. Post-contrast images revealed an enhancing mass in the medial right posterior parietal lobe that was 2.6 cm anterior-posterior, 2.3 cm transverse, and 2.1 cm cephalocaudal diameter. White matter edema was noted on imaging.

**Figure 3:**
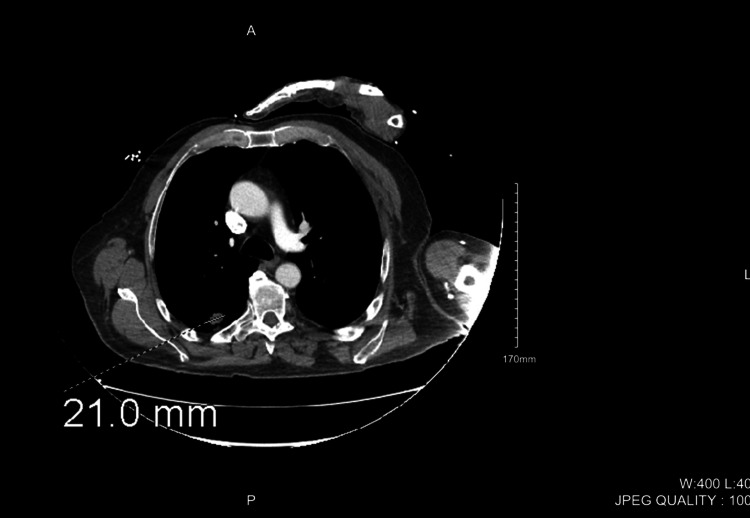
CT of the lung showing one of two apical right lower lobe tumors CT: computed tomography

**Figure 4:**
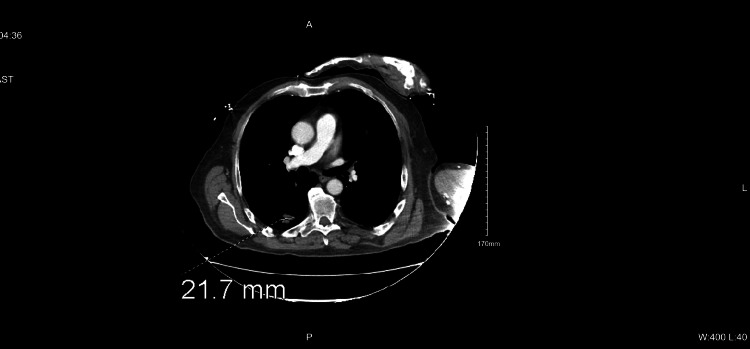
CT of the lung showing the second of two apical right lower lobe tumors CT: computed tomography

Figure 3 and Figure 4 depict the CT of the chest with contrast that was taken. Two masses were found in the apical right lower lobe, each approximately 2.2 cm in diameter. No pleural effusions were noted on imaging. 

The patient underwent an interventional radiology-guided lung biopsy of pulmonary masses, which pathology diagnosed as malignant melanoma. Immunochemistry staining of the lung masses was positive for HMB-45, Melan-A, S-100, and SOX-10 and negative for TTF1, NAPSIN-A, and CK7. 

The patient's wife stated that between 2007 and 2010 he had a suspicious skin lesion over his right temple. The patient underwent a Mohs excision procedure, which yielded negative results. The patient did not receive any further follow-up.

**Figure 5:**
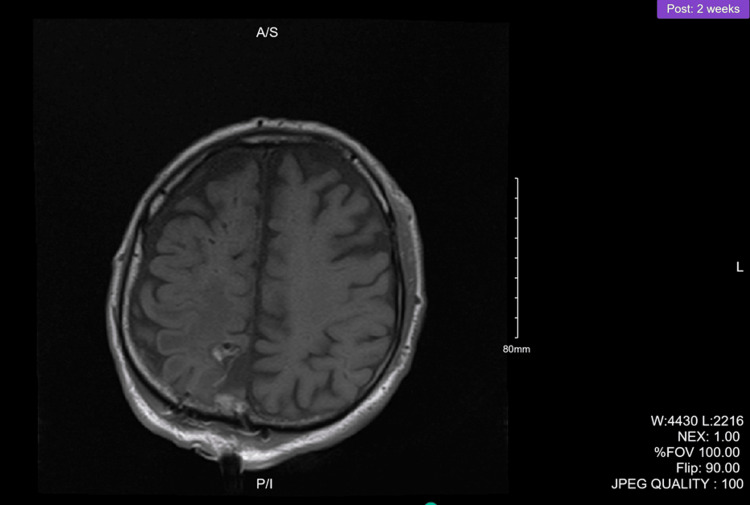
Postoperative brain MRI coronal view MRI: magnetic resonance imaging

**Figure 6:**
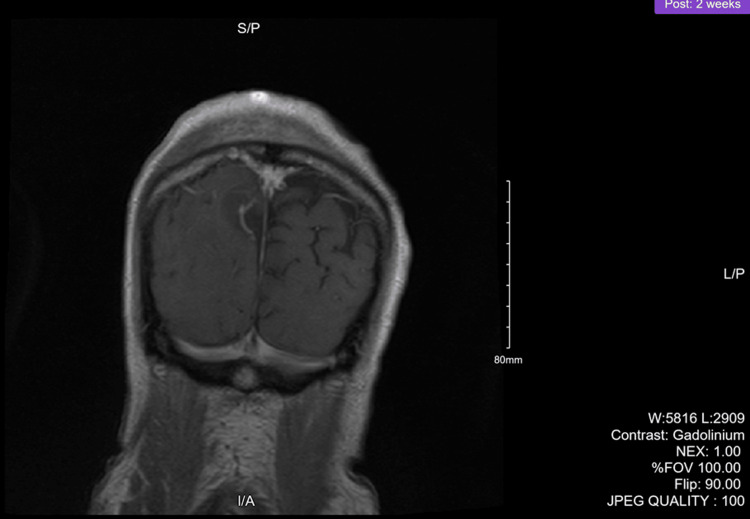
Postoperative brain MRI sagittal view MRI: magnetic resonance imaging

Craniotomy and mass resection of brain metastases were executed by our neurosurgeons. Preoperatively, the patient was continued on Keppra and Decadron. Figure 5 and Figure 6 depict the images from a brain MRI series taken on postoperative day 1. Findings were post-surgical changes with moderate vasogenic edema but no gross herniations or hemorrhages. 

The patient's postoperative CT of the head without contrast was negative for hemorrhage but showed a small pneumocephalus. Following postoperative care inpatient, he was sent home in a stable condition. Due to insurance, the patient was scheduled for treatment of his lung melanoma via surgical resection and radiation at their home hospital in Palm Springs. We contacted the patient at three months post-operation; the patient was doing well as he prepared for adjuvant chemotherapy. Unfortunately, the patient was lost to follow-up despite repeated attempts made at months 6, 9, and 10. 

## Discussion

Literature review

Search Criteria 

A literature search was performed in PubMed for free articles in English within the last 10 years. The following search terms were applied: ("melanoma of unknown primary" OR "PMML" OR "melanoma of the lung" OR "Pulmonary melanoma" OR "lung melanoma" OR "primary pulmonary melanoma") AND ("diagnosis" OR "detection" OR "screening" OR "evaluation" OR "identification" OR "assessment" OR "characteristics" OR "clinical features" OR "symptoms" OR "manifestations" OR "signs" OR "prognosis" OR "outcome" OR "survival" OR "prognostic factors" OR "prognostic markers" OR "treatment" OR "therapeutics" OR "management" OR "interventions" OR "therapy" OR "surgery" OR "chemotherapy" OR "immunotherapy" OR "targeted therapy" OR "radiation therapy" OR "prevention").

**Figure 7:**
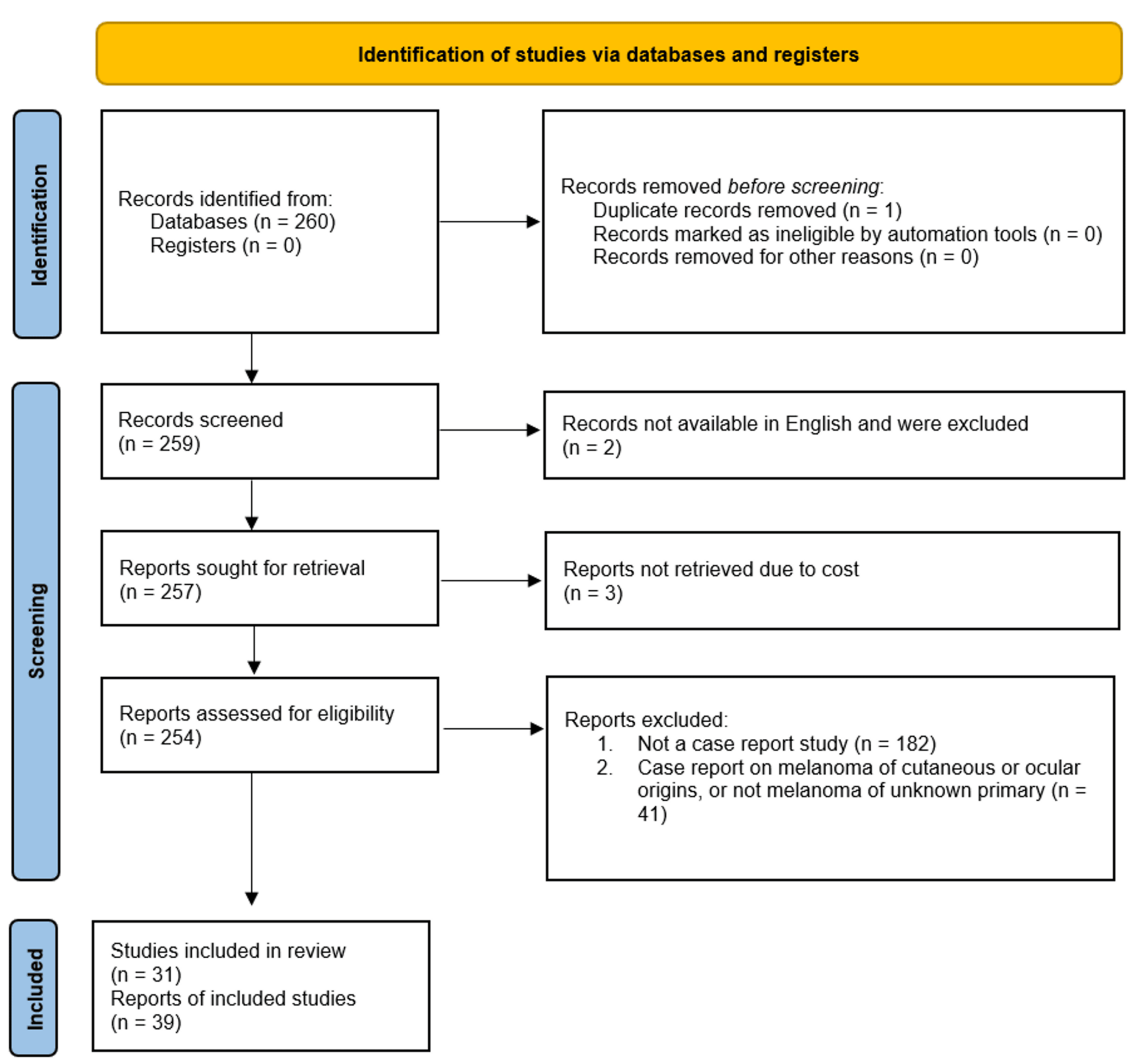
PRISMA diagram outlining the selection of recent case reports to include in the review PRISMA: Preferred Reporting Items for Systematic Reviews and Meta-Analyses

The initial search yielded 259 studies, after duplicates were removed. Articles were reviewed independently by three reviewers, TR, DR, and GK. The primary inclusion criteria were case reports of MUP, with the additional inclusion of cases of PMML, given our initial suspicion of PMML for our patient. The Preferred Reporting Items for Systematic Reviews and Meta-Analyses (PRISMA) diagram in Figure 7 below outlines the inclusion and exclusion criteria. The final number of studies was 31. Table 1 further highlights the inclusion and exclusion criteria used for publication selection.

**Table 1:** Inclusion and exclusion criteria

Inclusion criteria	Exclusion criteria
Case reports of melanoma of unknown primary within the last 10 years	Non-case reports (182)
	Case reports on melanoma of cutaneous or ocular origins or not melanoma of unknown primary (41)

Paper Review Process and Data Collection

Three reviewers (TR, DR, GK) extracted data, each publication was reviewed by two reviewers, and discrepancies were reviewed and unanimously agreed upon. Patient information, imaging modalities, biochemistry results, treatments, and outcomes were extracted for analysis. The information was then used for the statistical calculations by NR, detailed in our Results section.

Diagnosing MUP

Of the 31 papers, 14 cited Dasgupta et al.'s [5] original exclusion criteria when determining MUP [6-19]: (1) evidence of previous orbital exenteration or enucleation; (2) evidence of previous skin excision, electrodesiccation, cauterization, or other surgical manipulation of a mole, freckle, birthmark, paronychia, or skin blemish; (3) evidence of metastatic melanoma in a draining lymph node with a scar in the area of skin supplying that lymph node basin; and (4) lack of a non-thorough physical examination, including ophthalmologic, anal, and genital exam.

For each included publication, a thorough history and physical assessment of the patient was utilized by study authors to rule out melanoma of a known primary source. CT was the standard for 31/39 cases, with the most frequent exception being a prior positron emission tomography (PET) scan with increased uptake suggestive of cancer. The other two exceptions were due to a previous incorrect diagnosis of gastrointestinal stromal tumor and one unexplained, possibly due to tumor location in the temporalis muscle [10,20].

Pujani et al. [7] also refer to the recommendations for initial staging examinations of MUP suggested by Schlagenhauf et al. [21]. Recommendations included a detailed examination of areas drained by lymph nodes, chest X-ray, CT, ultrasound of relevant areas, and MRI of the brain for all MUP cases. 

The authors also reference possible explanations suggested by Clerico et al. [22] for the incidence of MUP including (1) malignant transformation of ectopic melanocytic/nevus cells and (2) complete regression of the primary melanoma after metastasis. A case report by Tahara et al. [23] provided possible evidence for the theory of complete regression with a case report of amelanotic melanoma of the nail that had regressed prior to MUP diagnosis with lymph node metastasis. 

Finally, Sirvan et al. [17] suggest that all previous excision material be re-examined for melanoma to further assist in definitively diagnosing MUP. Since melanoma is characteristically a cutaneous or ocular manifestation, diagnosis of an atypical site for melanoma should utilize substantial supportive evidence to rule out other possible explanations.

Epidemiology of MUP

**Figure 8:**
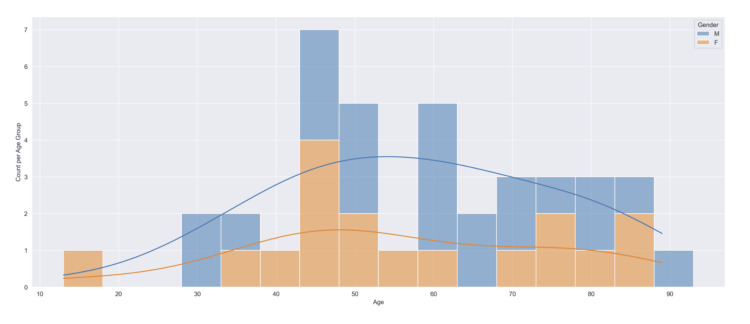
Distribution of counts of male and female sex within each age group (original work)

Our review included a total of 39 case reports with instances of MUP occurring in countries throughout the globe. Including this patient case report, six presented in the lung, and 12 presented in lymph nodes, five in the central nervous system, six in the gastrointestinal tract, four in the liver, three in the muscle, and three in the bone. Forty-five percent of data was derived from the United States, Ireland, and Japan combined. The mean age of our patient population was 57.5, with a range of 13-89. The male-to-female ratio was 1:1.3, with 22 male and 17 female patients (Figure 8). We examined various environmental exposures that may have influenced each patient's likelihood of acquiring cancer. Factors included smoking, alcohol, drugs, prior suspicious skin lesion, UV exposure, prior history of cancer, family history of cancer, physical trauma, chronic medications, and comorbidities. Chronic medications of note included prolonged steroid or immunosuppressant use. Comorbidities included chronic health conditions such as atrial fibrillation, chronic obstructive pulmonary disease, asthma, hypertension, diabetes mellitus, myocardial infarction, or recent history of infection such as pneumonia or acute pancreatitis. The greatest instance of MUP occurred in patients with prior smoking history and comorbidities, accounting for 17.95% of the population each. Around 7.69% of patients reported a family history of cancer, and 59% of patients had no reported environmental exposures.

Symptoms at presentation

Each case presented as a unique constellation of symptoms which we broadly categorized as gastrointestinal, musculoskeletal, neurological, pulmonary, palpable mass, incidental discovery, B-symptoms, skin findings, and either no symptoms at presentation or not reported. Twenty-one percent of patients presented with gastrointestinal symptoms, including nausea, vomiting, abdominal pain, or anorexia. Ten percent presented with musculoskeletal symptomatology, including back pain, shoulder pain, or chest wall pain. Neurologic presentation included numbness/tingling, seizures, saddle anesthesia, urinary incontinence, weakness, headache, or visual disturbances, accounting for 15% of patients. Pulmonary symptoms included cough, chest pain, hemoptysis, and dyspnea, contributing to 10% of initial presentations. Approximately 31% of patients first presented with a growing palpable mass, in some cases causing secondary symptoms such as dysphonia or dysphagia. Patients presented with a wide array of palpable mass locations, including in the inguinal region, axilla, temporal region, breast, and heel. Twenty-one percent of cases had an initial presentation of B-symptoms, which we categorized as weight loss, night sweats, anorexia in the absence of abdominal symptoms, and fevers. A small minority of patients, 3%, presented with a skin finding (non-melanoma) of pain, itchiness, ulceration, or erythema. In 10% of cases, patients did not present with any specific symptoms but were referred to the hospital after an incidental lab or imaging finding. In an additional 15% of cases, patients either did not present with any symptoms or no symptoms were reported. 

Diagnostics and subsequent biomarkers 

Imaging to diagnose MUP was broad, given that the melanotic lesion may have presented in any anatomical location. Common diagnostic modalities included biopsy (97.44%), CT (79.49%), PET-CT (56.41%), MRI (43.59%), esophagogastroduodenoscopy (12.82%), ultrasound (25.64%), ophthalmological examination (20.51%), dermatological examination (15.38%), colonoscopy (12.82%), X-ray (10.62%), and ears-nose-throat examination (7.69%). Less common methods included bone scan, computed tomography angiography (CTA), gynecological examination, lumbar puncture, mammography, magnetic resonance angiography (MRA), and thoracentesis, employed for 2.56% of patients each. All patients underwent multiple forms of imaging to fully survey the body for additional lesions and a potential primary site. Out of 39 patients, 38 underwent biopsy for diagnosis. The single patient who did not was solely because their risk of bleeding was too high. Following biopsy, 69% of patients had positive biomarker findings that supported the diagnosis of melanoma. The biomarkers reported included S-100 (49%), HMB-45 (44%), Melan-A (38%), BRAF (21%), MITF, V-9, CD79a, vimentin, TTF1, Ki67, P-16, c-kit, and SOX-10. The majority of patients tested positive for multiple biomarkers, but either 31% of patients diagnosed with melanoma did not test positive for any biomarkers or the results were simply not reported. 

Treatment and outcomes

**Figure 9:**
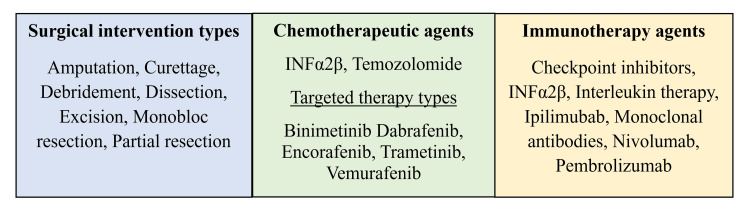
Treatment modality by category

Patients diagnosed with MUP were treated with various combinations of therapies tailored to their unique presentation. Treatment modalities included a combination of chemotherapy, radiation, surgery, immunotherapy, non-chemotherapeutic or immuno-modulatory medications, and palliative treatments. The only non-chemotherapeutic or immuno-modulatory medication used were antibiotics for one patient who presented with an ulcerative and necrotic lesion of the skin. Given that cancer treatment is often a process with multiple stages of intervention, we broke down patient treatments into primary and secondary interventions. Table 2 provides additional details for the primary treatment modality, including specific chemotherapeutic, immuno-modulatory, and other medical regimens, types of surgeries performed, and percentages of patients receiving each treatment. Table 3 provides the same information for the secondary treatment modality. Figure 9 specifies subclasses of the different types of treatment modalities listed in Table 2 and Table 3. Patients who had no intervention reported or did not undergo intervention or palliative measures accounted for 10% of patients in the primary treatment category. Forty-nine percent of patients required only primary treatment and did not undergo secondary treatment. For the primary treatment method, the most common intervention was surgery, with 62% of patients undergoing some form of procedure. For the secondary treatment method, immunotherapy was the most common, accounting for 38% of patients.

**Table 2:** Primary treatment modality by count

Treatment type	Counts	Percentage of population
Surgery	24	61.54%
No treatment reported	4	10.26%
Immunotherapy	4	10.26%
Radiation	4	10.26%
Medication	1	2.56%
Palliative care	2	5.13%
Chemotherapy	2	5.13%

**Table 3:** Secondary treatment modality by count

Treatment type	Counts	Percentage of population
No treatment reported	19	48.72%
Immunotherapy	15	38.46%
Chemotherapy	6	15.38%
Radiation	3	7.69%
Palliative care	1	2.56%
Surgery	1	2.56%

**Figure 10:**
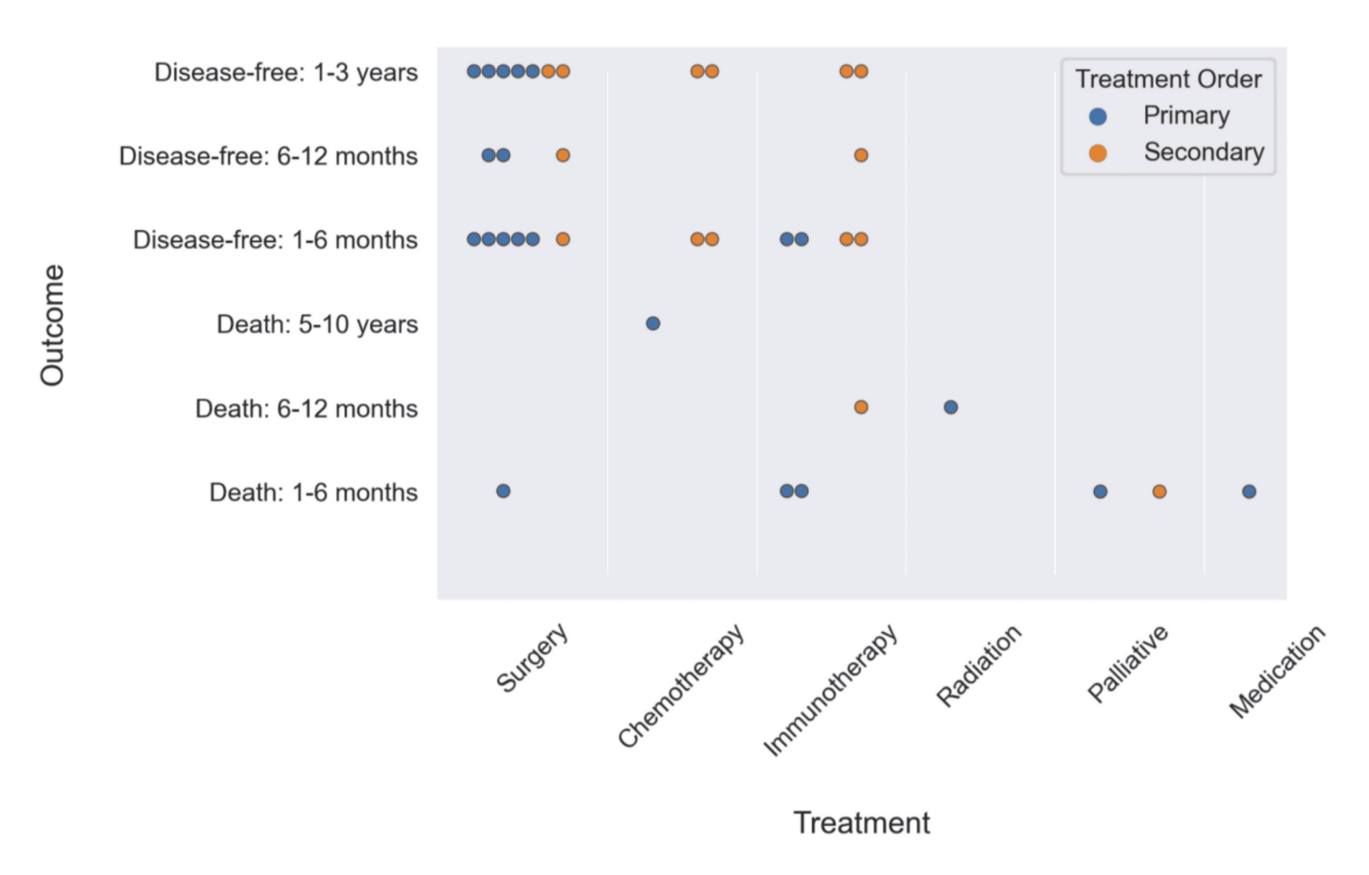
Primary and secondary treatment modality versus outcome of death or disease-free period (original work)

A wide range of data was collected on patient outcomes. To standardize this data, we broke up outcomes into general categories of death, disease-free period, and not reported. Within both the death and disease-free period categories, we split the data into bins of 1-6 months and 6-12 months. Additionally, within the disease-free period, we have a bin of 1-3 years, and within the death category, we have a bin of 3-5 years (no deaths were reported within 1-3 years). Unfortunately, nearly half of our data (49%) did not have any reported outcomes. As a result, our sample size for recorded outcomes is only 20 cases. For each of the 20 cases with reported outcomes, a graphic is provided in Figure 10 that shows counts of death and disease-free period for each defined time frame, for both primary and secondary treatment modality.

PMML

Given our patient's incidental finding of malignant melanoma of the lung without an identified skin origin, we initially suspected the diagnosis of PMML. This extremely rare non-epithelial neoplasm is characterized by its aggressive nature, high recurrence rate, and poor prognosis. A recent systematic review by Paliogiannis et al. [24] notes PMML's main characteristics: predominantly in males (64.4%), diagnosed around age 60, no clear link to smoking, and rarely preceded by a history of excised skin lesions that were unrelated to subsequently diagnosed PMML (8.3%). Immunostaining was commonly positive for S-100 (92.7%), HMB-45 (94.3%), and Melan-A (85.7%). Many cases (67.3%) presented with at least one distant visceral metastasis at the time of diagnosis [24]. Additional literature reports that PMML tumors often present as endobronchial lesions, leading to symptoms such as cough, hemoptysis, pneumonia, lobar collapse, or atelectasis. Moreover, approximately 30% of cases are incidentally detected on chest radiography, underscoring the diagnostic complexities of this condition [25]. 

The exact pathogenesis of PMML remains a subject of debate. Several hypotheses have been proposed to elucidate its occurrence, including the migration of benign melanocytes during embryogenesis, the presence of melanocytes and melanocytic proliferation in adjacent anatomical structures, and the possibility of a common embryological origin among the larynx, esophagus, and lungs, facilitating the migration of melanocytes [1,26]. Additionally, spontaneous regression of previous skin lesions and melanogenic metaplasia in the submucosa have been suggested as potential mechanisms contributing to the development of PMML [27]. 

Although there are no gold standard criteria for diagnosing PMML, existing literature recommends the following clinicopathologic diagnostic criteria listed in Table 4.

**Table 4:** Diagnostic criteria for PMML PMML: primary malignant melanoma of the lung References: [26-30]

Clinicopathologic diagnostic criteria for PMML
Absence of a history suggestive of a previous melanoma, including a history of excision or electrocauterization of skin, mucosal, or ocular lesions
Absence of demonstrable melanoma outside the thorax at the time of surgery
Presence of a solitary lung mass or nodule
Tumor morphology is compatible with that of a primary tumor
No evidence at autopsy of a primary melanoma elsewhere
Immunohistochemical staining for S-100 and HMB-45 and possibly by electron microscopy
Evidence of junctional change with nesting of cells beneath the bronchial epithelium or spindle cells arranged in fascicles
Invasion of the intact bronchial epithelium by melanoma cells in an area without epithelial ulceration

The remaining articles covered in this review were included within the figures seen above [31-45].

## Conclusions

To be classified as MUP by Dasgupta et al.'s exclusion criteria 2 or as PMML by the above-listed inclusion criteria 1, the patient must not have had a previous skin excision of a suspicious mass that could be a cutaneous melanoma. While our patient did undergo a Mohs excision of his right temple lesion, the results were negative for melanoma, so this patient does in fact fulfill these criteria. However, because this patient was found to have two lung masses at the time of discovery and not a "solitary lung mass or nodule," he does not meet the diagnostic criteria for PMML on the basis of inclusion criteria 3. Our patient instead meets the diagnostic criteria for MUP.

Through this case report and supporting literature review, we highlight two rare presentations of melanoma: MUP and PMML. While our patient ultimately meets the diagnostic criteria for the former, it is worthwhile to address the pathogenesis, presentation, and diagnostics of each. We detail a case in which MUP presented itself in the lung without an identified skin lesion, bringing light to the nuances separating the diagnoses of MUP and PMML. Despite limitations in sample size and data heterogeneity, our study highlights the diverse presentation and disease course of MUP, including overlap with PMML, calling for further studies to patient outcomes for rare melanoma presentations. Further investigations are necessary for a more enhanced understanding of diagnostic and therapeutic approaches for both MUP and PMML.

## References

[1] Yunce M, Selinger S, Krimsky W, Harley DP (2018). Primary malignant melanoma of the lung: a case report of a rare tumor and review of the literature. J Community Hosp Intern Med Perspect.

[2] Mada PK, Garibay M, Graham RL (2023). Primary malignant melanoma of the lung (PMML); a case report. Cureus.

[3] Scott JF, Gerstenblith MR Melanoma of unknown primary. Noncutaneous Melanoma.

[4] Agrawal CR, Talwar V, Tayal J, Babu Koyyala VP, Goyal P (2018). Primary pulmonary melanoma: an unexpected diagnosis. Indian J Pathol Microbiol.

[5] Dasgupta T, Bowden L, Berg JW (1963). Malignant melanoma of unknown primary origin. Surg Gynecol Obstet.

[6] Doyle C, O'Sullivan B, Watchorn RE, Eustace K (2023). Melanoma of unknown primary: a case series. Ir J Med Sci.

[7] Pujani M, Hassan MJ, Jetley S, Raina PK, Kumar M (2017). Malignant melanoma presenting as a mediastinal malignant melanoma presenting as a mediastinal unknown primary origin?. Turk Patoloji Derg.

[8] Yuan Z, Guo HY, Lu WT (2023). Report on a case of liver-originating malignant melanoma of unknown primary. Open Life Sci.

[9] Bankar S, Patkar S, Desai S, Shrikhande SV (2015). Unusual presentation of melanoma of unknown primary origin: a case report and review of literature. J Cancer Res Ther.

[10] Dalle Carbonare M, Goh MX, AlshiekhAli Z, Howlett D (2017). Metastatic melanoma of unknown primary in the temporalis muscle. BMJ Case Rep.

[11] Kim BC, Kang HK, Kim YS, Haw S, Kim HS, Kang J (2023). A rare case of endobronchial melanoma of unknown primary. Respir Med Case Rep.

[12] Suzuki T, Kusumoto S, Iida S, Tada T, Mori F (2014). Amelanotic malignant melanoma of unknown primary origin metastasizing to the bone marrow: a case report and review of the literature. Intern Med.

[13] Tanaka K, Tomita H, Hisamatsu K, Hatano Y, Yoshida K, Hara A (2015). Acute liver failure associated with diffuse hepatic infiltration of malignant melanoma of unknown primary origin. Intern Med.

[14] Kiedrowicz M, Halczak M, Kładny J, Królicki A, Maleszka R (2015). Melanoma of unknown primary origin coexisting with early-onset multifocal basal cell carcinoma. Postepy Dermatol Alergol.

[15] Andrianandrasana NO, Randrianarisoa RM, Navoly P, Ranaivoson MA, Vololontiana HM, Rafaramino F (2023). Melanoma of unknown primary origin with skeletal muscle metastasis: a case report. J Med Case Rep.

[16] Grech A, Mercieca N, Calleja-Agius J, Abela R (2020). Metastatic malignant melanoma of unknown primary in temporalis muscle. J Surg Case Rep.

[17] Sirvan SS, İhsan Eren H, Kurt Yazar S, Günenç AC, Yeşilada AK, Irmak F, Tuncel D (2019). Approach to patients with malignant melanoma of unknown primary origin. Sisli Etfal Hastan Tip Bul.

[18] Takahashi T, Sugita S, Kagaya H, Morita T (2020). Surgically treated gastric melanoma of unknown primary: a case report from a 10-year survivor. Pathol Int.

[19] Jin Y, Ran C, Li F, Cheng N (2020). Melanoma of unknown primary in the pancreas: should it be considered primary?. BMC Surg.

[20] Cortellini F, Marasco G, Renzulli M, Vasuri F, Ricciardiello L (2021). Gastric melanoma of unknown primary. J Gastrointestin Liver Dis.

[21] Schlagenhauff B, Stroebel W, Ellwanger U (1997). Metastatic melanoma of unknown primary origin shows prognostic similarities to regional metastatic melanoma: recommendations for initial staging examinations. Cancer.

[22] Clerico R, Bottoni U, Paolino G, Ambrifi M, Corsetti P, Devirgiliis V, Calvieri S (2012). Melanoma with unknown primary: report and analysis of 24 patients. Med Oncol.

[23] Tahara J, Kaku Y, Takimoto Ito R (2020). Amelanotic melanoma of the nail apparatus with regression previously diagnosed as melanoma of unknown primary site with a lymph node metastasis: a case report. J Dermatol.

[24] Paliogiannis P, Fara AM, Pintus G (2020). Primary melanoma of the lung: a systematic review. Medicina (Kaunas).

[25] Deng S, Sun X, Zhu Z (2020). Primary malignant melanoma of the lung: a case report and literature review. BMC Pulm Med.

[26] Huang D, Gou W, Zhang S, Gou Y, Dong X (2023). Primary pulmonary melanoma with brain metastasis: a case description and literature analysis. Quant Imaging Med Surg.

[27] Hwang KB, Hwang KE, Jung JW (2015). Primary pulmonary malignant melanoma: an unexpected tumor. Tuberc Respir Dis (Seoul).

[28] Kyriakopoulos C, Zarkavelis G, Andrianopoulou A, Papoudou-Bai A, Stefanou D, Boussios S, Pentheroudakis G (2017). Primary pulmonary malignant melanoma: report of an important entity and literature review. Case Rep Oncol Med.

[29] Peng J, Han F, Yang T, Sun J, Guan W, Guo X (2017). Primary malignant melanoma of the lung: a case report and literature review. Medicine (Baltimore).

[30] Allen MS Jr, Drash EC (1968). Primary melanoma of the lung. Cancer.

[31] Averbukh LD, Mavilia MG, Aujla AK (2019). Secondary gastrointestinal melanoma of unknown origin: a case report of a rare entity. Cureus.

[32] Eltawansy SA, Panasiti R, Hasanien S, Lourdusamy D, Sharon D (2015). Metastatic malignant melanoma of the inguinal lymph node with unknown primary lesion. Case Rep Med.

[33] Liu ET, Hsu CH, Chang DH (2024). Management of advanced stage IV melanoma of unknown primary origin with multiple visceral metastases. Asian J Surg.

[34] Nguyen V, Aboulenain S, Mohammed S, Perez Parra S (2022). A case of metastatic CNS melanoma of unknown primary presenting with seizures. Case Rep Med.

[35] Tanaka M, Matsumura M, Okudela K (2019). Pulmonary melanocytic nevus - a case report with a mutation analysis of common driver oncogenes. Pathol Int.

[36] Chen L, Newby C, Fakhri N, Lammle M (2021). Metastatic melanoma of unknown origin mimicking neurofibromatosis. Radiol Case Rep.

[37] Tang Y, Su YC (2019). Multiple bone metastases from an amelanotic melanoma of unknown primary origin. Kaohsiung J Med Sci.

[38] El Haj NI, Hafidi S, Karam R, Boubia S, Karkouri M, Ridai M (2021). Thoracic metastasis of malignant melanoma of unknown primary: a case report and literature review. Int J Surg Case Rep.

[39] Mremi A, Goodluck G, Sadiq A, Lodhia J (2021). Metastatic malignant melanoma of unknown primary site to the brain: a case report. Int J Surg Case Rep.

[40] El-Tani Z, Duc C, Gluecker T, Cottier O (2016). Intramammary metastatic melanoma of unknown primary origin in a 58-year old patient: a case report. J Med Case Rep.

[41] Baniak N, Podberezin M, Kanthan SC, Kanthan R (2017). Primary pulmonary/pleural melanoma in a 13 year-old presenting as pleural effusion. Pathol Res Pract.

[42] Matsumoto K, Kikuchi K, Kikuyama T (2021). Disseminated bone marrow carcinomatosis due to malignant melanoma of unknown primary origin. Intern Med.

[43] Rieth JM, Bowen RC, Milhem MM, Boldt HC, Binkley EM (2021). Presumed melanoma of unknown primary origin metastatic to the choroid mimics primary uveal melanoma. Case Rep Ophthalmol.

[44] Wang W, Liu J, Kang Y (2023). Diffuse hepatic infiltration of malignant melanoma of unknown primary origin. Asian J Surg.

[45] Wright JP, Geevarghese SK (2021). Primary heptaic melanoma or melanoma of unknown primary?. Am Surg.

